# Corrigendum: Recurrent Convolutional Neural Networks: A Better Model of Biological Object Recognition

**DOI:** 10.3389/fpsyg.2018.01695

**Published:** 2018-09-11

**Authors:** Courtney J. Spoerer, Patrick McClure, Nikolaus Kriegeskorte

**Affiliations:** Medical Research Council Cognition and Brain Sciences Unit, University of Cambridge, Cambridge, United Kingdom

**Keywords:** object recognition, occlusion, top-down processing, convolutional neural network, recurrent neural network

In the original article, there was a mistake in Table 1 as published. A small error was made in the calculation of the number of parameters in the networks. This caused the original figures to be inflated in all models by fewer than 100 parameters. The corrected Table 1 appears below.

**Table 1:** Brief descriptions of the models used in these experiments including the number of learnable parameters and the number of units in each model.

**Model**	**Kernel size**	**No. Features**	**No. parameters**	**No. units**
B	3 × 3	32	9,898	40,970
B-F	3 × 3	64	38,218	81,930
B-K	5 × 5	32	26,794	40,970
BT	3 × 3	32	19,114	40,970
BL	3 × 3	32	28,330	40,970
BLT	3 × 3	32	37,546	40,970

Additionally, heading 3.1 was incorrectly titled as “Recognition of Sights under Debris”. The correct heading is “Recognition of Digits under Debris”.

Also, there was an error in the text. When McNemar's test was used to test for a significant difference in multiple digit recognition tasks, we treated each digit as an independent sample. However, the probabilities for correctly identifying multiple digits in the same image are not independent. Therefore, we fail to meet the assumption of independence between samples required for McNemar's test. Instead, a test that corrects for dependence between samples should have been used, such as the variation on McNemar's test proposed by Durkalski et al. (2003). Using this corrected test produces a marginal difference in the results and leads to no change in the significance of the tests.

A correction has been made to *Results, Recognition of Multiple Digits, Paragraphs 1 and 2*:

To examine the ability of the networks to handle occlusion when the occluder is not a distractor, the networks were trained and tested on their ability to recognize multiple overlapping digits. In this case, when testing for significance, we used a variant of McNemar's test that corrects for dependence between predictions (Durkalski et al., 2003), which can arise when identifying multiple digits in the same image.

When recognizing three digits simultaneously, recurrent networks generally outperformed feedforward networks (Figure 7), with the exception of BT and B-K where no significant difference was found [χ^2^(1, *N* = 30, 000) = 3.82, *p* = 0.05]. All other differences were found to be significant (FDR = 0.05). The error rates for all models are shown in Table 4. A similar pattern is found when recognizing both four and five digits simultaneously. However, in both four and five digit tasks, all pairwise differences were found to be significant, with B-K outperforming BT (Figure 7). This suggests that, whilst recurrent networks generally perform better at this task, they do not exclusively outperform feedforward models.

The authors apologize for these errors and state that this does not change the scientific conclusions of the article in any way.

The original article has been updated.

## Conflict of interest statement

The authors declare that the research was conducted in the absence of any commercial or financial relationships that could be construed as a potential conflict of interest.
